# Thermodynamics and electronic structure of adsorbed and intercalated plumbene in graphene/hexagonal SiC heterostructures

**DOI:** 10.1038/s41598-024-53067-3

**Published:** 2024-02-05

**Authors:** Simone Brozzesi, Paola Gori, Daniel S. Koda, Friedhelm Bechstedt, Olivia Pulci

**Affiliations:** 1 Department of Physics and INFN, University of Rome Tor Vergata, Via della Ricerca 1, I-00133 Rome, Italy; 2 Department of Industrial, Electronic and Mechanical Engineering, Roma Tre University, Via della Vasca Navale 79, I-00146 Rome, Italy; 3 Lawrence Livermore National Laboratory, 7000 East Ave, L-367, Livermore, CA 94551 USA; 4 Institut für Festkörpertheorie und -optik, Friedrich-Schiller-Universität, Max-Wien-Platz 1 07743 Jena, Germany

**Keywords:** Electronic properties and materials, Surfaces, interfaces and thin films

## Abstract

Graphene-covered hexagonal SiC substrates have been frequently discussed to be appropriate starting points for epitaxial overlayers of Xenes, such as plumbene, or even their deposition as intercalates between graphene and SiC. Here, we investigate, within density functional theory, the plumbene deposition for various layer orderings and substrate terminations. By means of total energy studies we demonstrate the favorization of the intercalation versus the epitaxy for both C-terminated and Si-terminated 4H-SiC substrates. These results are explained in terms of chemical bonding and by means of layer-resolved projected band structures. Our results are compared with available experimental findings.

## Introduction

The enormous interest in graphene^1^ has rapidly induced the interest in other two-dimensional (2D) group-IV materials with a hexagonal honeycomb structure, belonging to the family of so-called Xenes, for their properties and their possible applications^2^. The Xenes silicene, germanene, and stanene are however fundamentally different from graphene due to their buckled geometry and their fundamental gap at the *K* point of the Brillouin zone (BZ), which is induced by the spin-orbit coupling (SOC). The heaviest lead-derived Xene, plumbene, was also predicted with a honeycomb structure in its freestanding version. Since the other Xenes, with weaker SOC, are topological insulators and, as 2D systems also quantum spin Hall (QSH) insulators, the topological properties of plumbene, with its huge SOC, became of central interest. Contradictory results^3,4^, somewhat in dependence on layer buckling, can be found in literature. Even a spin Chern insulator has been predicted theoretically^5^.

The recent epitaxy of plumbene^6^ has ended the experimental realization of all group-IV cousins of graphene with a honeycomb structure^2^. Its synthesis was realized on a metallic substrate, more precisely a Pd_1-x_ Pb_x_ dilute alloy film on a Pd(111) bulk, as many Xenes before on Ag(111) or Au(111) substrates^7^. However, in the case of epitaxial graphene-like layers, besides metallic surfaces, also surfaces of insulators have been used to grow this flat 2D Dirac material^8^. Further, the deposition of Xenes as van der Waals (vdW) bonded monolayers on quasi-freestanding graphene layers on top of hexagonal SiC(0001) has been suggested^9^.

Moreover, there are several reports on intercalation of SiC-supported graphene by various atoms^10,11^. The interaction between graphene and adsorbed or intercalated alkali-metal, alkaline earth-metal and hydrogen atoms has some analogies to the well-known graphite intercalation^12^. The intercalation was originally considered as a strategy to produce quasi-freestanding graphene layers by decoupling the buffer layer from the SiC(0001) substrate^13–15^. Recently, the intercalation method has been used or suggested to use to produce Xene layers (see e.g. Refs.^16,17^). Epitaxial graphene on SiC(0001) is also perfectly suited for plumbene. Indeed, experiments trying to intercalate Pb under a graphene layer on SiC have been performed^18–22^. The intercalation of plumbene has been experimentally demonstrated for a ($6\sqrt{3}\times 6\sqrt{3})R30^\circ$ carbon buffer layer, sometimes also called zero layer graphene (ZLG), grown on Si-terminated 6H-SiC(0001) surfaces^21^. Such a graphene layer is commonly prepared by Si sublimation via annealing of a hexagonal SiC substrate^23^. The preference of Pb intercalation versus Pb adsorption has been confirmed by thermodynamic studies^24^.

In the present paper we investigate the geometry, the energetics and resulting electronic properties of intercalated versus adsorbed plumbene layers in/on a graphene monolayer on top of a hexagonal SiC(0001) or SiC(000$\bar{1}$) substrate by means of density functional theory (DFT) and a superlattice approach. Together with the optimized structures, a phase diagram describing the different Pb overlayers with varying density and position is derived to identify the favored surface arrangement in the thermodynamic equilibrium. The electronic structure is especially investigated by applying a layer-resolved band structure technique. Special attention is paid to the influence of layer arrangement, bonding and atomic density on the modifications of the Dirac bands of graphene like, e.g., a possible band gap opening.

The paper is structured as follows. We first describe the theoretical and computational methods used to study the heterostructures. We then present our results for the geometry, the electronic band structure and the energetic stability of the various models used to describe the intercalation of Pb under graphene on SiC. We also investigate the case in which a graphene buffer layer is introduced. Finally, conclusions are given.

## Theoretical and numerical methods

### Total energy and electronic structure calculations

The structural and electronic properties of the various layered systems are investigated within the DFT framework as implemented within the Quantum Espresso package^25,26^. The exchange and correlation (XC) functional derived within the generalized gradient approximation (GGA) is chosen according to Perdew, Burke and Ernzerhof (PBE)^27^. Van der Waals (vdW) interaction is treated by means of the vdW-DF method^28–30^. The electron-ion interaction is described by normconserving, fully relativistic pseudopotentials. The outermost *s* and *p* electrons are treated as valence electrons and the electronic configurations $2s^2$ $2p^2$ (C), $3s^2$ $3p^2$ (Si) and $6s^2$ $6p^2$ (Pb) are taken into account for the group-IV atoms. A plane-wave cutoff of 120 Ry and a 4$\times$4$\times$1 $\textbf{k}$-points grid are used during the geometry optimizations. The structural relaxation gives rise to residual Hellmann-Feynman forces below 2.5 meV/Å and total energy changes smaller than 1 meV/atom. Because of the heavy Pb atoms, SOC is then added to the ground state of total energy studies and in the electronic structure calculations. We observed a substantial influence of the SOC on the energies of the Pb-containing systems. The neglect of SOC in other studies^24^ with the argument that SOC does not affect the results for ground states^31^ may be only reasonable for atomic geometries. Total energy calculations were performed using a shifted $2\times 2 \times 1$ uniform Monkhorst-Pack $\textbf{k}$-point mesh^32^ and 160 Ry plane wave energy cutoff.

### Modelling of layered structures

**Figure 1:**
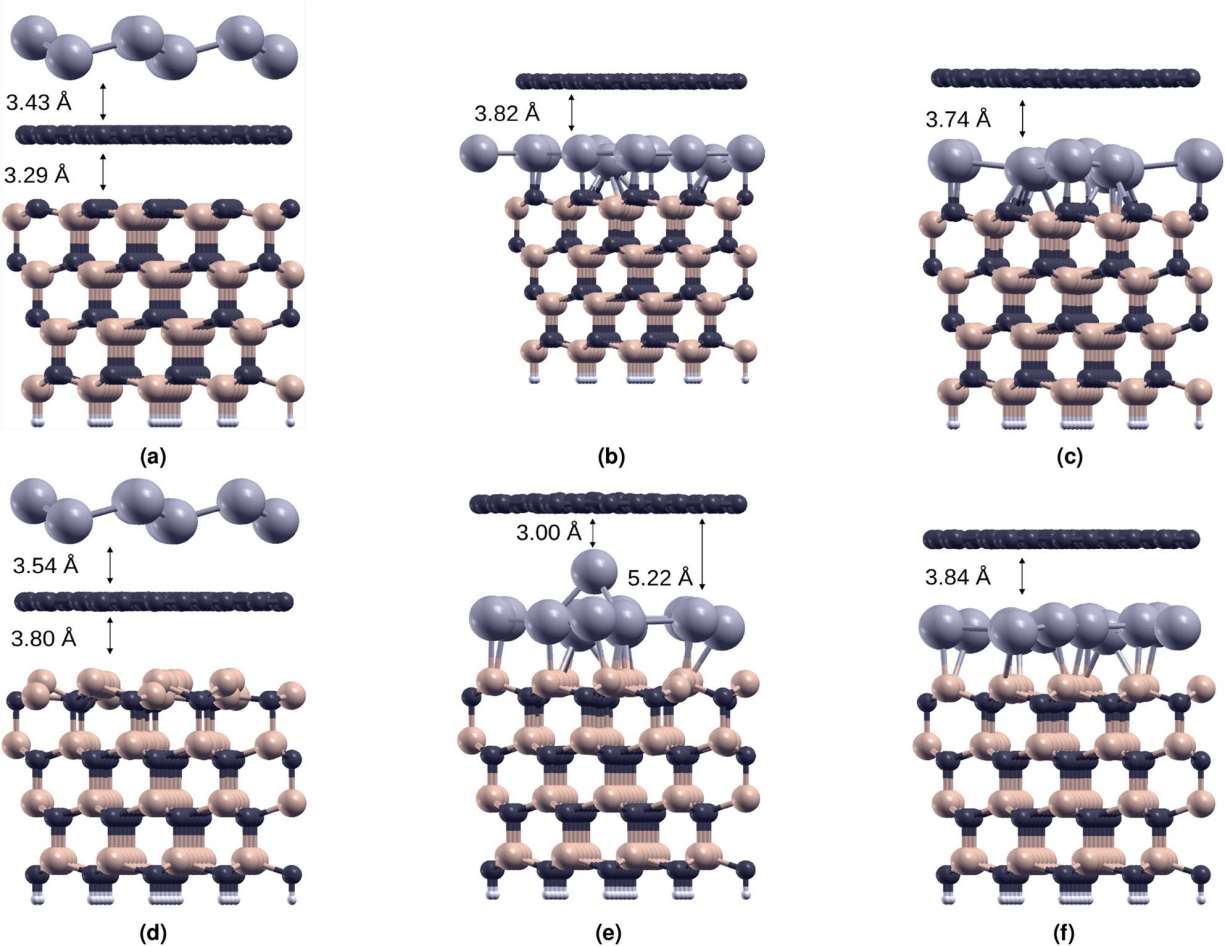
Atomic geometries of the studied systems: graphene and Pb on C-terminated SiC (**a**–**c**), and on Si-terminated SiC (**d**–**f**). In (**c**) and (**f**) one less Pb atom (13 instead of 14) in the $(\sqrt{7} \times \sqrt{7})R19.1^\circ$ cell is considered.

In order to study all layered systems under consideration we apply the supercell approach. For the modelling of the basic graphene/SiC heterostructures in Fig. 1, for which we study the intercalation or adsorption of Pb atoms, we choose four SiC bilayers, i.e., two 4H-SiC bilayers, stacked in [000$\bar{1}$] (Fig.1a–c) or [0001] (Fig.1d–f) directions. The bottom Si or C atomic layers are passivated by H atoms. To characterize the translation symmetry of the overlayers, the Wood notation^33^ is used to classify the two surface Bravais lattices in comparison to the original $1 \times 1$ surface.

**Figure 2:**
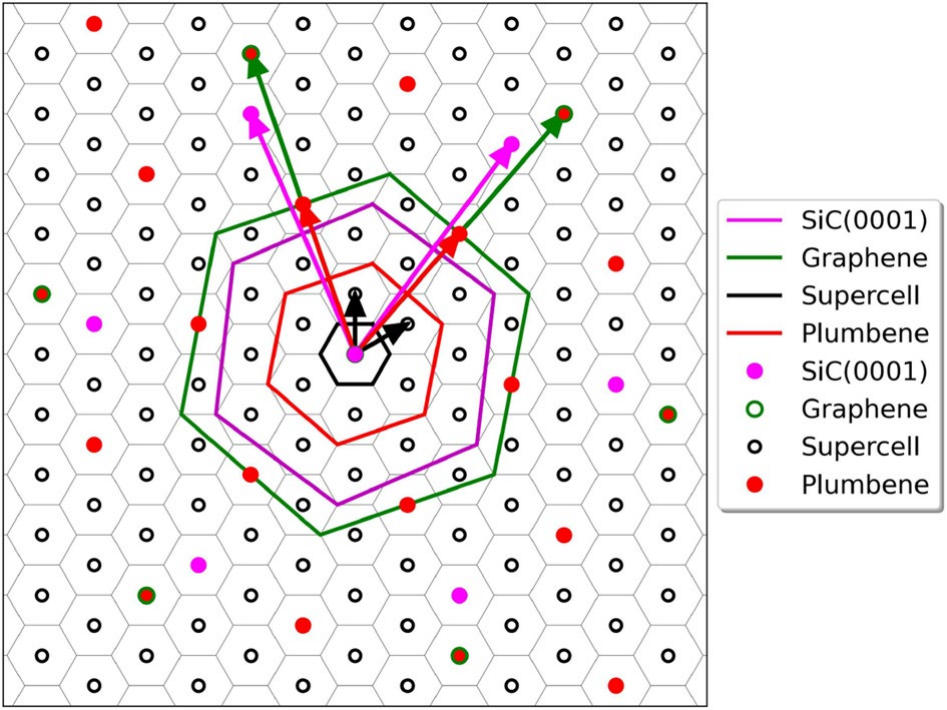
Arrangement of the BZs of the supercell of the heterostructure and of the $1 \times 1$ unit cells of the constituent layers. The reciprocal basis vectors, the BZs of the primitive $1 \times 1$ cells of graphene (green), plumbene (red) and SiC(0001) (magenta hexagons) and the BZ of the supercell (black and grey hexagons) are shown. The size and the orientation of the BZs follow the geometry description that has been exposed in the text.

Experimentally, on a 6H- or 4H-SiC(0001) substrate, an initial carbon layer with honeycomb lattice develops with a 2D $(6\sqrt{3} \times 6\sqrt{3})R30^\circ$ superstructure^34^. Superstructures with $(6 \sqrt{3} \times 6\sqrt{3})R30^\circ$ reconstructions lead to too many atoms in a supercell, 864 (108 $\times$ 4 layers) SiC atoms $+$ 108 H atoms $+$ 216 C atoms in the graphene-like overlayer, resulting in a total of 1188 atoms. Such a number cannot be easily handled numerically. For that reason, we decided to investigate smaller cell sizes of the 2D superstructures. In literature^35–37^, to model graphene on Si- and C-terminated SiC surfaces, frequently a $(\sqrt{3}\times \sqrt{3})R30^\circ$ lateral unit cell model is applied, despite the $(6\sqrt{3}\times 6\sqrt{3})R30^\circ$ periodicity observed experimentally. If a lattice mismatch between graphene and the SiC lattices of about 8$\%$ is accepted, the $(\sqrt{3}\times \sqrt{3})R30^\circ$ cell can be combined with a $2\times 2$ graphene cell^36^. We assumed pseudomorphic adaption between the graphene overlayer and the SiC substrate. This is generated by application of the coincidence lattice method^38^. We found that a substrate with a 4H-SiC(000$\bar{1}$) $(\sqrt{19} \times \sqrt{19})R23.4^\circ$ surface unit cell and a graphene overlayer with a $(2\sqrt{7} \times 2\sqrt{7})R19.1^\circ$ translational symmetry nearly fulfills a coincidence condition with a small tensile strain in the overlayer of about 3.5%. The hexagonal lattice constants of the two separated systems are 13.41 Å (SiC) and 13.05 Å (ideal $(2\sqrt{7} \times 2\sqrt{7})$ Gr). The advantage of the resulting Gr/SiC heterostructure is that a plumbene layer with a $(\sqrt{7} \times \sqrt{7})R19.1^\circ$ hexagonal unit cell (lattice constant 12.96 Å) fits reasonably well to the SiC substrate. For the heterostructure, we used the substrate optimized lattice constant. This results in a tensile strain of about 4.2% in an adsorbed plumbene overlayer. The number of C or Si atoms in the uppermost rather flat atomic layer of the 4H-SiC(0001) or (000$\bar{1}$) surface, 19 in a unit cell, and that of the C atoms in the corresponding graphene unit cell, here 56, make that only a few bonds between substrate and graphene are formed. For a more clear depiction of the heterostructure composition in relation to its constituent layers, a reciprocal space map has been generated, as shown in Fig. 2. The BZ of the supercell arrangement, along with the BZs of individual layers, each related to its respective 1$\times$1 unit cell arrangement, is presented. The supercell’s basis vectors are delineated in black. In green, the basis vectors of graphene are represented, while magenta corresponds to those of SiC, and red to those of 2DPb. The figure clearly elucidates the relative orientation of the various layers. A judicious selection of rotation angles results in a coincidence lattice where each K-point in every BZ maps onto a corresponding *K*-point in the supercell’s BZ.

Several distances between graphene and SiC have been considered in the starting configurations for atomic relaxation. In particular, distances of the order of the sum of the vdW radii, and below, have been investigated.

## Results: Pb on Graphene/SiC(0001) and Graphene/SiC(000$\bar{1}$)

### Atomic geometries

We have studied three classes of systems: 4H-SiC(0001) and (000$\bar{1}$) substrates with one graphene and one plumbene layer, as displayed in Fig. 1, and a SiC(0001) substrate with a graphene buffer layer, a graphene layer, and plumbene. This last configuration describes the situation where ZLG is formed. We discuss first the two cases of 4H-SiC(0001) and (000$\bar{1}$) substrates with one graphene and one plumbene layer, whereas the last results section is devoted to the ZLG case.

The relaxed atomic geometries for intercalation or epitaxial deposition of a plumbene-like layer in the presence of one monolayer of graphene are displayed in Fig.1a–c (for C-terminated SiC surface in the Gr/SiC heterostructure) and Fig.1d–f (for the Si-terminated case). Following the coincidence lattice method^38^ we added (before atomic relaxation) a twisted ($\sqrt{7} \times \sqrt{7})R19.1^\circ$ plumbene overlayer with 14 Pb atoms in the supercell, which almost fits to the original graphene layer with respect to the hexagonal lattice constant and the rotation angle. The number of the much smaller carbon atoms in the $(\sqrt{7} \times \sqrt{7})R19.1^\circ$ cell is by a factor 4 larger. The different filling factors can be explained by the different covalent radii $r_{{\textbf {Pb}}}= 1.47$ Å and $r_{{\textbf {C}}} = 0.77$ Å^39^ of the Pb and C atoms, respectively. The ratio $(r_{{\textbf {Pb}}}/r_{{\textbf {C}}})^2$ = 3.64 of the corresponding areas is, in fact, close to the factor 4.

We have studied different arrangements and atomic densities of the plumbene-like layer, as shown in the three subfigures Fig. 1a–c, (for C-terminated SiC) and Fig. 1d–f (for Si-terminated SiC). For C-terminated SiC substrate, the adsorbate situation, Fig. 1a, clearly indicates a vdW-bonded plumbene overlayer with a buckling of $\Delta = 0.84$ Å, strongly indicating the $sp^3$ bonding between the Pb atoms, and an average distance of 3.43 Å to the vdW-bonded graphene, which, in turn, has a distance of 3.29 Å from the C-terminated 4H-SiC(000$\bar{1}$) substrate. Similarly, also in the Si-terminated case, Fig. 1d, graphene lies above the substrate, but at a larger distance of about 3.80 Å. Such configurations represent local minima on the total energy surface, after relaxing the structure starting from a graphene-SiC distance equal to the sum of the vdW radii. The graphene distance from the SiC surface is larger in the Si-terminated case, consistently with the larger vdW radius of Si as compared with C. Interestingly, the buckling $\Delta = 0.38$ Å of the last C-Si bilayer of the C-terminated substrate is significantly reduced as compared to its bulk value of 0.63 Å. This fact also indicates the adaption of the SiC surface to the overlayers and the strain minimization.

The situation is completely different in the intercalated structures displayed in Fig. 1b (with 14 Pb atoms) and Fig. 1c (with 13 Pb atoms) as well as in Fig. 1e and f. The plumbene-like layers in between graphene and SiC form strong covalent bonds to the substrate. This is also confirmed by the interlayer distance between the intercalated 2DPb layer and the surface of the SiC substrate. In the C-terminated case the interlayer distance is 2.24Å, and in Si-terminated case is 2.69Å. In both cases the distances are comparable with the sum of the covalent radii of the respective elements. This evidence is confirmed by the paper of Schädlich *et al. *^40^. Although their work is focused on a different reconstruction for the heterostructure, they measured a value of 2.72 Å for the Pb-SiC interlayer distance in the Si-terminated case, in presence of an overlying carbon buffer layer. Looking at the interlayer Pb-graphene spacing we observe an increase in this value after the intercalation both in C- (3.82Å vs 3.43Å) and Si-terminated case ($\approx 3.9$Å vs 3.54 Å). All these distances are comparable with the sum of the van der Waals radii of C, Pb and Si, confirming the full Van der Waals character of the interaction between the layers. It is worth to mention that the interlayer distance in these kind of heterostructure can be affected by tensile or compressive strain induced in the layer to achieve the commensurability of the supercells. Since the strain depends on the considered reconstruction, it can be concluded that there is a significant agreement with the experimental results in^40^. In dependence on the positions of the Pb atoms with respect to the dangling bonds of the SiC surface, vertical or non-vertical bonds are formed, which modify the buckling of the plumbene layer and tend to induce some roughness on it. In the Si-terminated case, the latter effect strongly depends on the Pb atomic density as illustrated in Fig. 1 e and f. The $(\sqrt{7}\times \sqrt{7})R19.1^\circ$ plumbene with 14 atoms in the unit cell leads to rather ordered intercalated Pb layers, with 6-fold and 5-fold Pb-rings, and one dumbbell, while the reduction of the number of atoms to 13 tends to destroy the rings structures giving rise to a rather disordered intercalate. In the C-terminated phase, both 13 and 14 Pb layers are disordered, and no honeycomb structure survives. The top views of the Pb layers are shown in the Supplementary Material (Figs. SM1, SM2).

### Energetics/thermodynamics

The total energies of various Pb, C, SiC arrangements in isolated, layered or multilayer systems discussed above are listed in Table 1. The quality of the underlying total energy calculations may be illustrated by the cohesive energy $E_{coh}$ = 2.08 eV/atom for bulk metallic Pb. This value is only slightly above the experimental value of $E_{coh} = 2.03$ eV/atom^41^. The direct comparison of the total energies in Table 1 for intercalated system Gr/Pb/SiC and the adsorbate systems Pb/Gr/SiC shows that Pb intercalation is clearly favored independent of the substrate termination. The difference in energy is 3.32 eV/$1 \times 1$ plumbene cell for C-termination, in qualitative agreement with other theoretical studies^24^. The reason is obvious from Figs. 1a and d. The bonding of the plumbene layer to the dangling bonds of the C-terminated SiC(000$\bar{1}$) substrate makes intercalation more favorable. The situation in the Si-terminated case is very similar. Only the energy gain of 2.84 eV per 1$\times$1 plumbene cell of intercalation vs. adsorption is somewhat smaller.

**Table 1:** Total energies and work functions (WF) results for all the heterostructures and their constituent systems. In the case of freestanding and peeled-off plumbene layers, the two reported values are ionization energies and electron affinities. Si-term refers to the Si-terminated SiC substrate, and C-term to the C-terminated case. PO stands for peeled-off layer, for which the isolated plumbene and graphene calculations have been performed extracting their geometry from the heterostructure.

System	E_tot [Ry]	WF (eV)
Pb gas phase (per atom)	− 140.3395	
Pb bulk metal (per atom)	− 140.4921	
Plumbene (freestanding)	− 1966.3089	4.36/3.83
Plumbene PO C-term	− 1966.2872	4.36/3.70
Plumbene PO Si-term	− 1966.2845	4.36/3.70
Graphene (freestanding)	− 674.8085	4.24
Graphene PO C-term	− 674.4350	4.55
Graphene PO Si-term	− 674.5196	4.51
SiC slab C-term	− 1581.2708	5.49
SiC slab Si-term	− 1581.9570	3.62
Pb/Gr/SiC 14 C-term	− 4222.3359	4.86
Gr/Pb/SiC 14 C-term	− 4224.0457	5.13
Gr/Pb/SiC 13 C-term	− 4083.4806	5.13
Pb/Gr/SiC 14 Si-term	− 4223.1916	3.30
Gr/Pb/SiC 14 Si-term	− 4224.6545	3.58
Gr/Pb/SiC 13 Si-term	− 4084.2662	3.73
Pb/Gr/ZLG/SiC Si-term	− 4897.3733	3.27
Gr/Pb/ZLG/SiC Si-term	− 4897.3870	3.25
Gr/ZLG/Pb/SiC Si-term	− 4898.5129	3.82

The influence of the density of the Pb atoms in a layer on the relationship between epitaxial and intercalated plumbene cannot be studied based alone on the total energies in Table 1. Rather, the thermodynamics is ruled by the grandcanonical thermodynamic potential $\Omega (T, A,\mu _{C}, \mu _{Si}, \mu _H, \mu _{Pb})$ instead of the (excess) free energy $F(T,A,N_C,N_{Si},N_H, N_{Pb})$^33^. Here, we consider fixed temperature $T = 0$ K, area *A* of the 2D systems, and fixed numbers $N_C$, $N_{Si}$, $N_H$ of carbon, silicon, hydrogen atoms. Only the number $N_{Pb}$ of lead atoms is varied. The particle exchange with a reservoir characterized by the chemical potential $\mu _{Pb}$ is allowed. The reservoirs described by the chemical potentials $\mu _C$, $\mu _{Si}$, and $\mu _H$ of the other species do not play a role. Hence, restricting our calculations to the variation of the number of Pb atoms, that is, keeping the number of C, Si and H constant, one may simply write

1 $$\begin{aligned} \Omega (\mu _{Pb}) = E_{tot}(N_{Pb})-\mu _{Pb}N_{Pb}-\sum _i \mu _i N_i , \end{aligned}$$

where *i*=C, Si and H. By taking as zero energy the total energy of the Gr/Pb/SiC heterostructures with 14 Pb atoms in the ($\sqrt{7}\times \sqrt{7})R19.1^{\circ }$ unit cells in Fig. 1b and e), we plot the variation $\Delta \Omega$ of $\Omega$ for the other heterostructures versus the variation of the chemical potential per atom, $\Delta \mu = \mu _{Pb} - \mu _{Pb}^{metal}$, with respect to that of bulk Pb metal $\mu _{Pb} = E_{tot}(\text {Pb metal}) = -140.4921$ Ry. For the grand thermodynamic potential variation in a $(\sqrt{7}\times \sqrt{7})R19.1^{\circ }$ unit cell it holds

2 $$\begin{aligned} \Delta \Omega (\mu _{Pb}) = E_{tot}(N_{Pb})-E_{tot}(14) - [E_{tot}(\text {Pb metal}) + \Delta \mu _{Pb}](N_{Pb} - 14) \end{aligned}$$

with $E_{tot}(14)$ as the total energy of an intercalated with 14 Pb atoms in the unit cell in Table 1.

**Figure 3:**
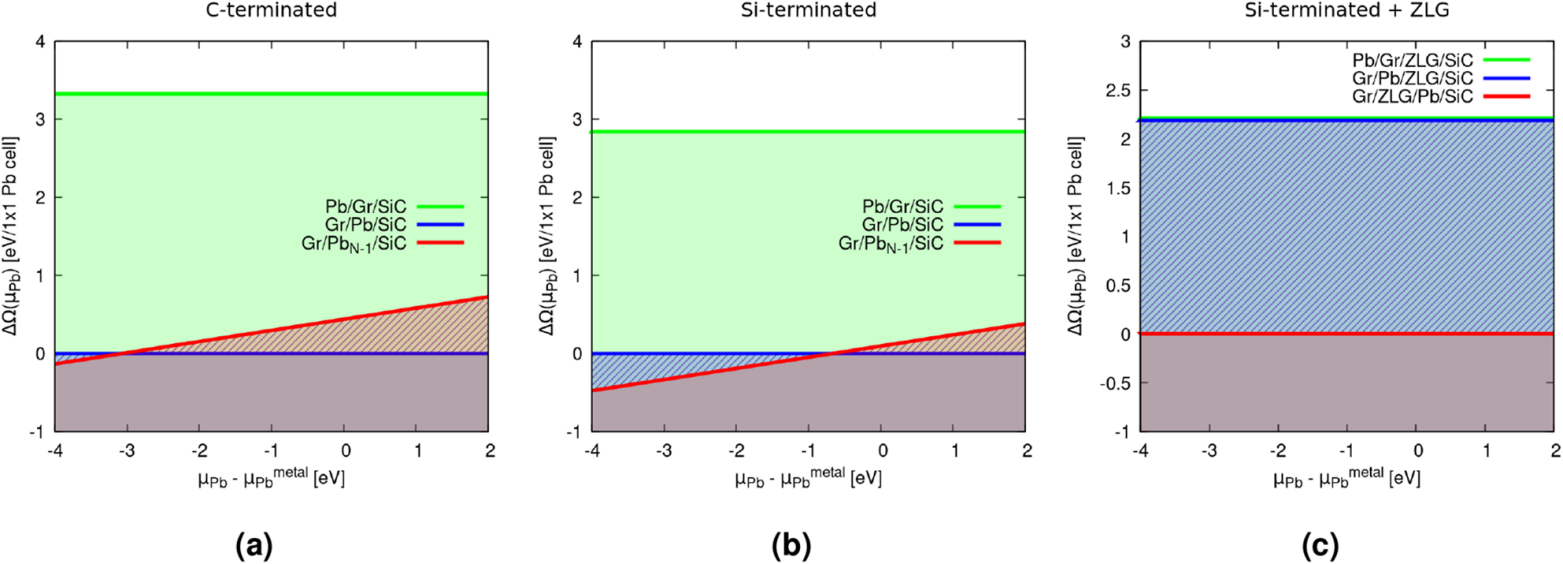
Grandcanonical thermodynamic potential difference $\Delta \Omega$ of the heterostructures evaluated for several substrates (**a**) C-terminated SiC(000$\bar{1}$) and (**b**) Si-terminated SiC(0001), and (**c**) Si-terminated SiC(0001) + ZLG. The difference $\Delta \Omega$ takes as a reference the thermodynamic potential $\Omega$ of the fully intercalated system with 14 Pb atoms in the $(\sqrt{7}\times \sqrt{7})R19.1^\circ$ cell for each substrate. It is plotted versus the difference between the chemical potential of an arbitrary lead reservoir $\mu _{Pb}$ and that of the bulk Pb metal $\mu _{Pb}^{metal}$.

The resulting phase diagrams are shown in Fig. 3a for the C-terminated SiC and in Fig. 3b for the Si-terminated one. The chemical potential $\mu _{Pb}$ is allowed to vary around $\mu _{Pb}^{metal}$, which describes Pb-rich growth conditions, where metal-like clusters may be formed on/in the system during the preparation process. The deposited Pb atoms may also form Pb carbides or Pb silicides, although these compounds are not very stable. The formation of such compounds on or in the heterostructures corresponds to a chemical potential limit $\mu _{Pb}^{metal}-\Delta H_f$, which may be identified as Pb-poor conditions. Thereby, the formation enthalpy $\Delta H_f$ would correspond to the energy gained when forming PbC or PbSi compounds.

The resulting phase diagrams for the two substrates Gr/SiC(000$\bar{1}$) in Fig. 3a and Gr/SiC(0001) in Fig. 3b show again that intercalation of the plumbene layer between graphene and the SiC bulk is energetically favored compared to the epitaxial plumbene on top of a Gr/SiC(000$\bar{1}$) (Fig. 3 a) or Gr/SiC(0001) (Fig. 3 b). Thereby, the atomic density of the intercalated Pb layer may vary in dependence of the chemical potential $\mu _{Pb}$ characterizing the Pb reservoir. As mentioned, we plot $\Delta \Omega$ in the range of chemical potentials around $\mu _{Pb} \rightarrow \mu _{Pb}^{metal}$, i.e., a Pb-rich situation where the Pb atoms may locally form metallic clusters. In the C-terminated case, the plumbene with 14 Pb atoms in a ($\sqrt{7}\times \sqrt{7})R19.1^\circ$ unit cell is the most favorable one for Pb-rich growth conditions (blue line in Fig. 3a). However, for a less Pb-rich growth situation, the intercalation with only 13 Pb atoms becomes favorable (red line), as expected. Interestingly, instead, in the Si-terminated case (Fig. 3b), we find that the less dense layer (13 Pb atoms) is the energetically favorite condition in a larger range of variation of $\mu _{Pb}$. This can be understood by looking at Fig. 1e, which shows the presence of a Pb atom in a dumbbell position, a clear sign that 14 Pb atoms do not manage to accomodate on the SiC(0001) Si-terminated surface in a flat atomic layer.

### Electronic structures

#### C-terminated SiC substrate

**Figure 4:**
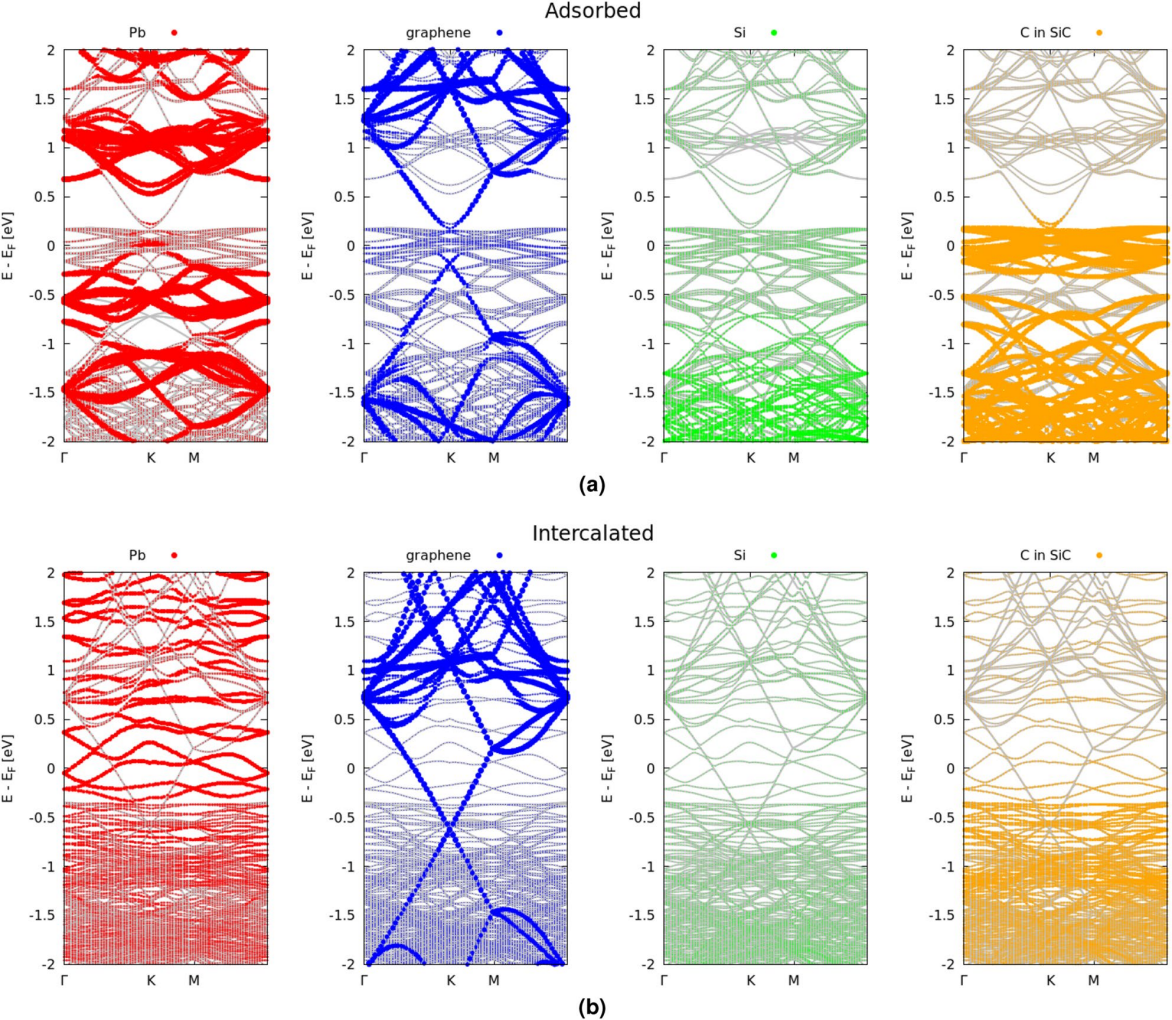
Band structure of the Pb/Gr/SiC(000$\bar{1}$) and Gr/Pb/SiC(000$\bar{1}$) (C-terminated SiC substrate) system (grey lines). The band structures projected onto the atoms in graphene (blue), plumbene layer (red) as well as the C (orange) and Si (green) atoms in the SiC substrate are indicated by different colors. To represent an atomic species the sum over the *s*, *p* and, when present, *d* orbitals is taken. The Fermi level is used as energy zero.

We first address the case of the interaction of plumbene with the Gr/SiC(000$\bar{1}$) substrate. The resulting band structures projected onto carbon atoms in the graphene layer, Pb atoms in the plumbene sheet as well as C or Si atoms of the SiC substrate are displayed in Fig. 4. The band structures are in line with the bonding behavior indicated in Fig. 1a. For the system that represents the adsorbate situation, the Dirac cones of plumbene and graphene at the corner *K* point of the BZ (Fig. 4a) survive and plumbene exhibits, in a way comparable with the findings for freestanding materials^42^, a SOC-induced gap of the order of 0.5 eV. Their mutual interaction and the interaction with the uppermost C layer of the SiC substrate is anyway visible by the band splittings of the upper Dirac cones of graphene and plumbene and the significant modification of the lower cones due to the C-derived dangling bond states of the SiC(000$\bar{1}$) substrate around the Fermi level $E_F$.

**Figure 5:**
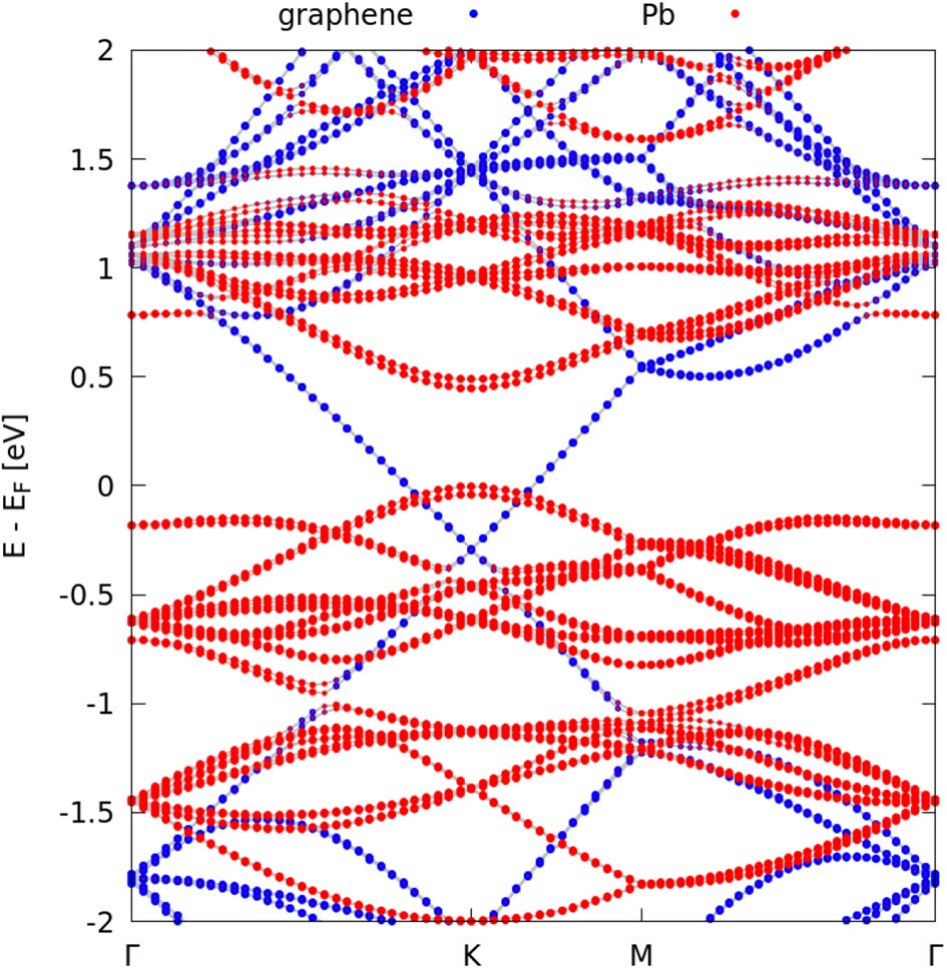
Band structure of the graphene-plumbene bilayer, peeled-off from the Gr/Pb/SiC heterostructure keeping the ($\sqrt{7}\times \sqrt{7}$)R19.1^∘^ cells and projected onto C (blue dots) and Pb (red dots) states. The Fermi energy is used as energy zero.

The significant influence of the uppermost C layer of SiC is underlined by the band structure, displayed in Fig. 5, of the plumbene/graphene bilayer when it is peeled off from the SiC substrate. Calculations have been performed in the same unit cell of the whole heterostructure, considering just the peeled-off Gr/Pb bilayer as it is relaxed on SiC, without any further relaxation. Some hybridized states are present in intermediate regions along the $\Gamma - K$, $K-M$ and $M - \Gamma$ paths. In general, the character of the states is well defined and discernible. The interaction between graphene and Pb results in a shift in energy of the cones states: graphene cones, in fact, are moved under $E_F$. Without the presence of the C-dangling bonds of SiC(000$\bar{1}$), the Dirac-like bands of graphene and plumbene are clearly visible. The Fermi level is now almost pinned at the top of the lower Pb-derived Dirac cone with a gap of about 0.45 eV to the upper cone. The Dirac point of graphene is shifted toward lower energies of about 0.25 eV indicating a minor filling of the upper Dirac cone with electrons by polarization effects.

The electronic structure of the total layered Gr/Pb/SiC(000$\bar{1}$) system with the intercalated plumbene layer in Fig. 4b is remarkably modified in comparison to the freestanding plumbene. Because of the strong bonding between Pb and C atoms from the uppermost SiC(000$\bar{1}$) substrate layer, but also interactions with the other layers, the uppermost valence bands around the Fermi level show contributions from plumbene, graphene and silicon atoms, but mainly from the uppermost C layer in SiC. The band structure with the gapped Dirac cones of freestanding plumbene is completely destroyed near the Fermi level. On the other hand, the graphene-derived Dirac cone practically remains uninfluenced compared to the freestanding situation. The vdW interaction and the electrostatics of the complete layered system Gr/Pb/SiC(000$\bar{1}$) only shift the Dirac point below the Fermi level by about 0.5 eV, approximately twice the shift observed in the peeled-off bilayer case in Fig. 5.

In Table 1 we also add the absolute positions of the Fermi level with respect to the vacuum level, i.e., the work function (WF), for the systems displayed in Figs. 4 and 5. We have to mention that the graphene value is slightly different from the one calculated for the freestanding $1\times 1$ graphene, with an increase of the WF of approximately 0.3 eV. The value in Table 1 is given for the ($2\sqrt{7}\times 2\sqrt{7})$R19.1^∘^ flat carbon layer, which is slightly compressively strained. In contrast, the ($\sqrt{7}\times \sqrt{7})$R19.1^∘^ plumbene layer is less affected by strain compared with the freestanding layer: the ionization energy and electron affinity are only shifted by less than 10 meV. As shown in Table 1, the WFs of Pb/Gr/SiC(000$\bar{1}$) and Gr/Pb/SiC(000$\bar{1}$) exhibit a drastic reduction of 0.63 and 0.35 eV, respectively, compared with the SiC(000$\bar{1}$) value, indicating lowering of the surface dipole (Fig. SM3) by the Gr/Pb or Pb/Gr overlayer. The freestanding Gr WF and the mid gap of freestanding plumbene are by 1.25 eV and 1.40 eV, respectively, below the WF value of the SiC(000$\bar{1}$) substrate. The much larger work function WF = 5.13 eV for Gr/Pb/SiC compared to WF = 4.86 eV for Pb/Gr/SiC is a further argument explaining why Pb intercalation is favored over Pb epitaxy: the occupied bands of Gr/Pb/SiC have a larger distance to the vacuum level, indicating that the band structure contribution to the total energy, the so-called band structure energy^33^, significantly contributes to the energy gain.

#### Si-terminated SiC substrate

**Figure 6:**
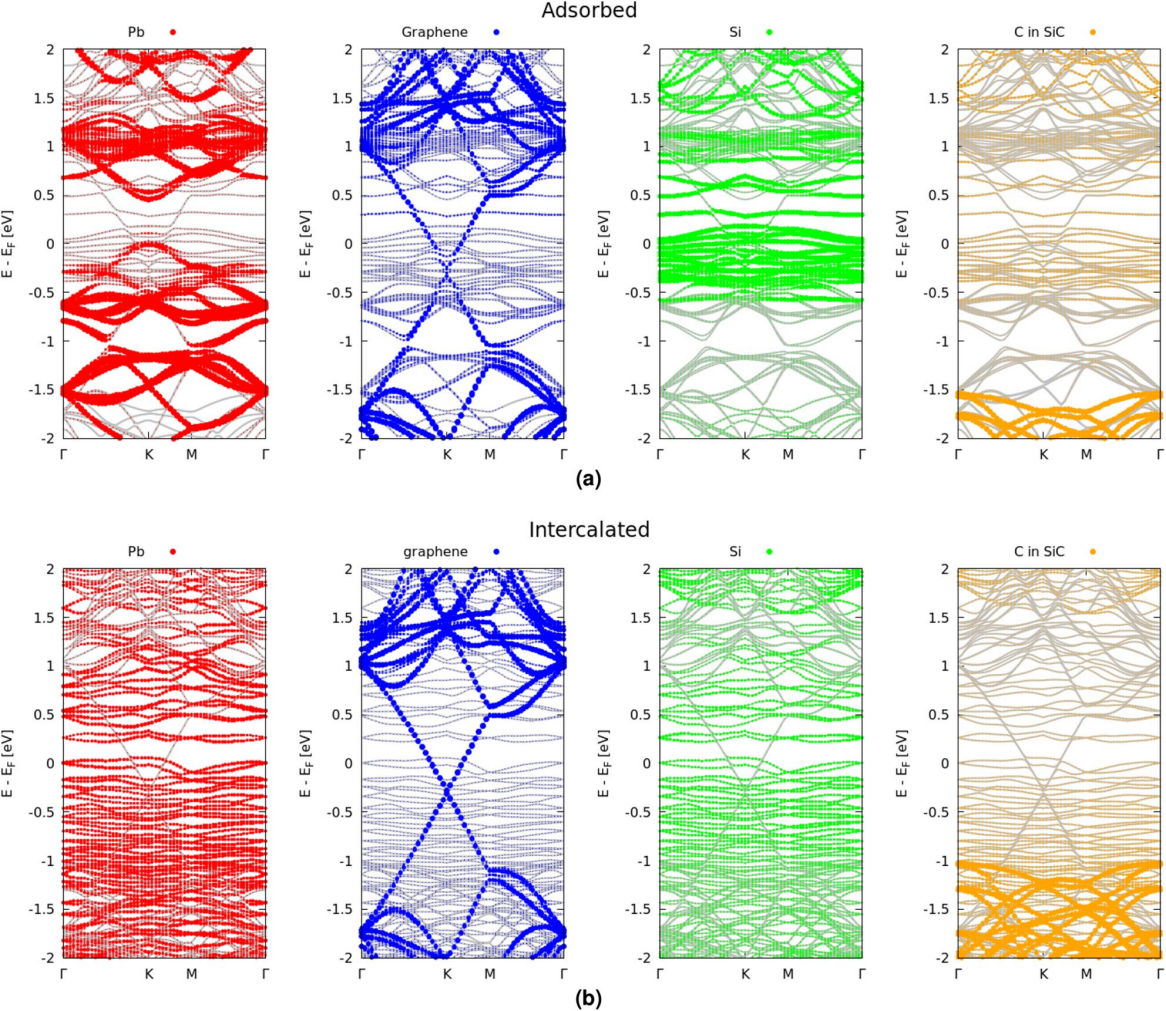
Band structure of the Pb/Gr/SiC(0001) and Gr/Pb/SiC(0001) (Si-terminated SiC substrate) system (grey lines). The band structures projected onto the atoms in graphene (blue), plumbene overlayer (red) as well as the C (orange) and Si(green) atoms in the SiC substrate are indicated by different colors. To represent an atomic species the sum over the *s*, *p* and, when present, *d* orbitals is taken. The Fermi level is used as energy zero.

We now discuss the interaction of plumbene with the Gr/SiC(0001) substrate. The electronic band structure is reported in Fig. 6. Also in this case, the band structures in Fig. 6a and b are consistent with the bonding behavior indicated in Fig. 1d and e. Before intercalation, traces of the Dirac cones of plumbene, with its SOC-induced gap, and of graphene are still visible in the proximity of the *K* point. After intercalation, however, the strong interaction between Pb and Si atoms of the SiC(0001) uppermost layer makes Pb Dirac cones disappearing and restores a quasi-freestanding behavior of Gr as already illustrated in Fig. 1e. The vdW interaction and the electrostatics of the complete layered system Gr/Pb/SiC(0001) shift the Dirac point approximately 0.3 eV below Fermi level.

As a consequence of the formation of Pb-Si bonding and antibonding states, the layered system (excluding the graphene states at the Dirac cones) shows in Fig. 6b an insulating behavior with a tiny indirect gap of 0.18 eV between Pb-Si-derived conduction and valence bands. However, the gap is bridged by Dirac states of graphene with a Dirac point in the valence bands.

Our electronic structure results in Fig. 6b are in qualitative agreement with experimental findings using scanning tunneling spectroscopy (STS)^21^ and photoemission spectroscopy^20^. The almost intact occupied Dirac cone of graphene is visible. Only an extremely small splitting of the Dirac cones of about 22 meV is observed. A splitting of about 300 meV in between occupied and empty Pb-derived states has been measured between shoulders in the STS spectrum and identified with the spin-orbit gap of plumbene at the *K* point. According to Fig. 6b this energy difference should be better related to Pb-Si bands. These facts illustrate that the graphene layer on top undergoes only a minor perturbation in contrast with the identification of the intercalated plumbene layer adsorbed to the substrate.

Concerning WFs, both in Pb/Gr/SiC(0001) and Gr/Pb/SiC(0001) there is a reduction compared with the SiC(000$\bar{1}$) case, that amounts to 0.32 and 0.04 eV, respectively. This again indicates a lowering of the surface dipole by the Gr/Pb or Pb/Gr overlayer, but of a smaller entity compared with the case of SiC(000$\bar{1}$) substrate. The reduction of the WF of Pb/Gr/SiC compared to the one of Gr/Pb/SiC is practically the same with the two SiC terminations: 0.28 eV for Si-terminated substrate and 0.27 eV for C-terminated one. In both cases, therefore, Pb intercalation is favored over Pb epitaxy.

## Results: Pb on Graphene/SiC(0001) with graphene buffer layer

**Figure 7:**
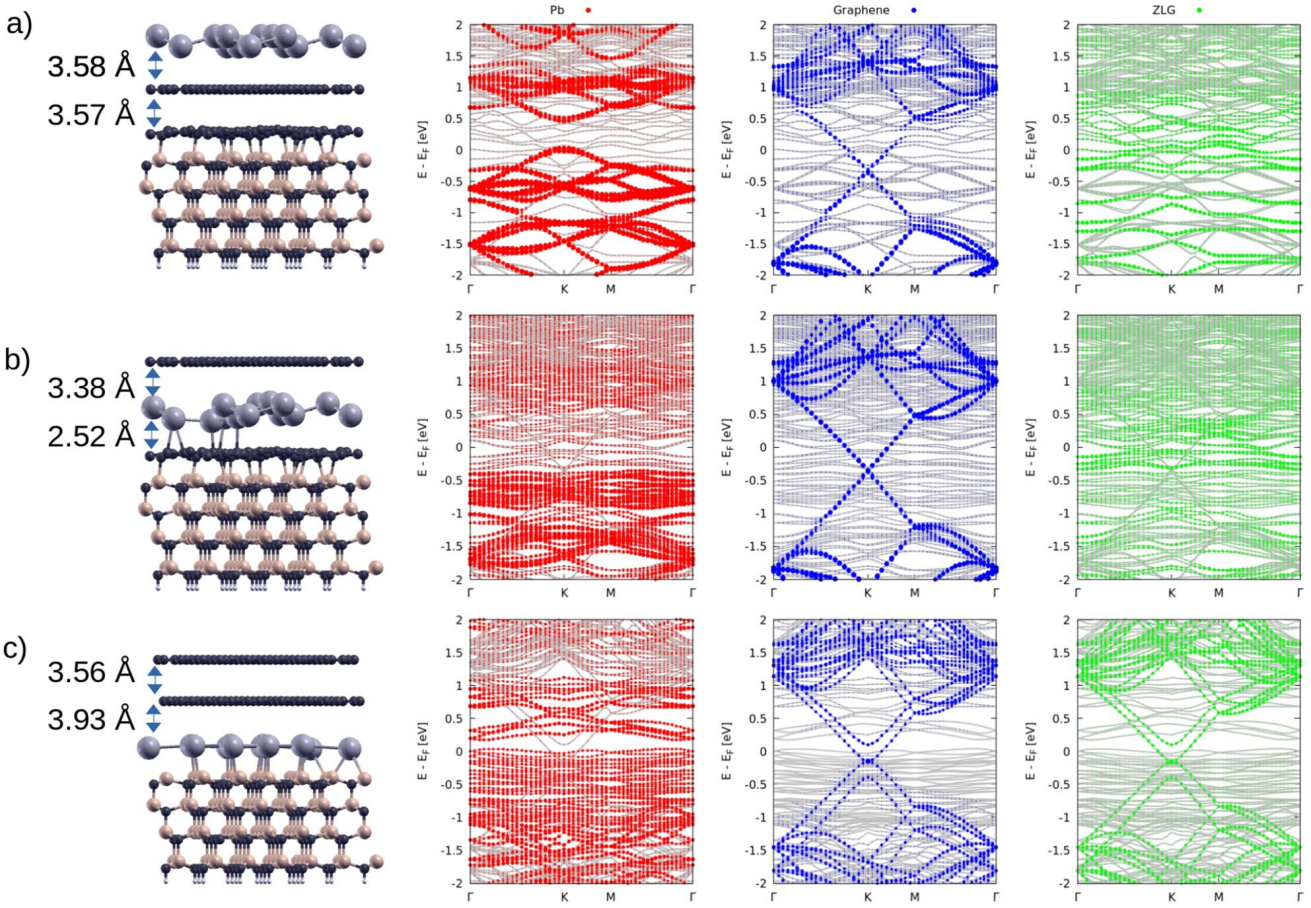
Side view of the crystal structures (left panel) and band structures (grey lines in right panels) for Pb on top of graphene on Si-terminated ZLG-SiC (**a**), Pb under graphene on Si-terminated ZLG-SiC (**b**) and Pb under bilayer graphene on Si-terminated SiC (**c**). The Fermi energy is used as energy zero. Red dots refers to states of Pb; blue dots to the upper graphene; green dots to the ZLG.

Finally, we have also considered the case where a ZLG is present. We show in Fig. 7 the SiC(0001) surface with an additional carbon layer on top of the substrate, the ZLG, which is strongly bonded without Pb intercalation in Fig. 7a and b (left panels). Without plumbene the graphene layer only exhibits a vdW interaction with the ZLG-covered SiC(0001) substrate. Deposition of plumbene on top leads to an almost freestanding adsorbate with the typical buckling. Consequently, the projected band structures in Fig. 7a (three right panels) indicate the survival of the electronic features of plumbene and graphene, whereas the ZLG-covered SiC substrate does not show neither a gap nor Dirac cones.

The plumbene intercalation between the two carbon layers in Fig. 7b gives rise to a bonding between plumbene and the ZLG, while the uppermost graphene layer only shows vdW interaction with a corresponding layer distance. Consequently, the Dirac cones of graphene survive locally, whereas the bonding between plumbene and ZLG and between ZLG and substrate indicate a metallic behavior. The plumbene intercalation between ZLG and SiC substrate in Fig. 7c shows, in principle, a similar geometrical situation as in the intercalated case in Fig. 1e, with a plumbene covalently bonded with the uppermost Si layer of SiC. Nevertheless, if compared with those in Fig.6b, the bands show striking differences due to the appearance of the band features of a graphene bilayer with Bernal stacking^43^ instead of the pure Dirac cones of isolated graphene. The two Dirac cones are modified near the Dirac point by band splitting and destroyed linear bands. Moreover, it can be observed that when Pb is on top of graphene, Fig. 7a, the Dirac point of graphene is located approximately at -0.38 eV from the Fermi energy, highlighting an n-doping in graphene induced by the plumbene layer and the underlying SiC + ZLG substrate. After the partial intercalation of Pb between graphene and the ZLG, Fig. 7b, the Dirac point of graphene is only slightly shifted upward, and is located -0.35 eV from the Fermi energy. In this scenario the plumbene layer is partially covalently bonded to ZLG, but still interacting with the above graphene, and the n-doping effect is still significant. Conversely, after full Pb intercalation, despite the modifications of the typical Dirac linear cone dispersion, the Dirac point of graphene is located -0.15 eV from the Fermi energy, with the n-doping level that is now quite lower. This suggests that the full intercalation of plumbene under graphene, in presence of a ZLG, efficiently decouples the graphene layer from the SiC substrate. This result finds experimental confirmation in the paper of Schädlich *et al. *^40^, in which photoelectron spectroscopy measurements reveal the formation of almost charge-neutral freestanding graphene after Pb intercalation. This evidence has been also experimentally observed by Hu *et al.*^44^.

The energetics of the three different systems with the positions of the plumbene layer in Fig. 7 (left panel) is also described in the phase diagram in Fig. 3c and Table 1. Also with the additional ZLG, intercalation of plumbene is favored over the adsorption. The comparison of the configurations displayed in Figs. 7a and 7b again shows a favorization of the intercalation, but only by 26 meV/1$\times$1 plumbene cell. However, the situation characterized by the occurrence of plumbene intercalation between ZLG and SiC and the formation of a graphene bilayer as overlayer represents the most favorable one with a huge energy gain of 2.188 eV/1$\times$1 plumbene cell. The work function in Table 1 are rather similar to the Gr/SiC(0001) case. Therefore, we conclude that the ZLG layer has only a weak influence of on the surface dipole.

## Summary and conclusions

By means of density functional theory we have investigated the deposition of 2D plumbene-like layers on graphene/4H-SiC substrates with C- or Si-termination. Even an additional carbon layer, the ZLG, has been studied. Especially the epitaxy and the intercalation of plumbene have been studied. The structural optimization of the layered systems with different sequence, density and termination has been combined with thermodynamic considerations. The structure and energetic results have been illuminated by studies of bonding, including van der Waals one, and the electronic structure. They are combined with results for subsystems, e.g. peeled off graphene/plumbene layers, and band structures projected onto atoms in the various layers.

The atomic geometries of plumbene on top of the Gr/4H-SiC(000$\bar{1}$) and Gr/4H-SiC(0001) systems as well as for the intercalated situation have been optimized starting from atomic arrangements obtained using the coincidence method. As a consequence, much larger lateral unit cells are considered, leading to much lower strains than in previous theoretical studies. The resulting formation energies clearly favor intercalation over epitaxy, for a wide range of chemical potentials of Pb atoms. The main reason is the formation of strong chemical bonds between the atoms of the intercalated plumbene and the atoms in the uppermost atomic layer of SiC.

The projected band structures, which allow for a layer and atom resolution, confirm the picture derived from the energetics. In the epitaxial case, the bands known from freestanding graphene and plumbene are less influenced by the vdW interaction than they are by some stronger interaction with the dangling bonds of the uppermost SiC layer, mainly in their upper occupied parts. In the energetically more favorable intercalation limit this observation holds for graphene on top with its almost intact Dirac cones. However, the strong covalent bonding of Pb atoms to the uppermost SiC layer atoms destroys the characteristic bands of plumbene around the *K* point in the BZ. Instead of SOC, the dominant interaction for gap opening now is the chemical bonding to the SiC substrate.

If a zero graphene layer is added, the favorization of intercalation versus adsorption of the Pb layer is still confirmed. However, interestingly, the Pb atoms like to intercalate the region between the ZLG and the SiC substrate. This intercalation tendency is due to the bonding of the Pb atoms to the substrate and results to a graphene bilayer on top of plumbene and almost completely decoupled from it.

### Supplementary Information

Supplementary Information.

## Data Availability

The data presented in this study are available on reasonable request from the corresponding author.
