# Supplementary material for: Thermodynamics and electronic structure of adsorbed and intercalated plumbene in graphene/hexagonal SiC heterostructures
Source: Sci Rep. 2024 Feb 5;14:2947. doi: 10.1038/s41598-024-53067-3 (PMC10844374; doi:10.1038/s41598-024-53067-3)
Supplement: Supplementary file 1 — Supplementary Information. [file 41598_2024_53067_MOESM1_ESM.pdf]

Supplementary Information for ”Thermodynamics and electronic structure of adsorbed and intercalated plumbene in graphene/hexagonal SiC heterostructures”

Simone Brozzesi,[1] Paola Gori,[2] Daniel S. Koda,[3] Friedhelm Bechstedt,[4] and Olivia Pulci[1]

[1]*Department of Physics and INFN, University of Rome Tor Vergata, Via della Ricerca 1, I-00133 Rome, Italy*

[2]*Department of Industrial, Electronic and Mechanical Engineering, Roma Tre University, Via della Vasca Navale 79, I-00146 Rome Italy*

[3]*Lawrence Livermore National Laboratory, 7000 East Ave., L-367, Livermore, CA 94551, U.S.A.*

[4]*Institut für Festkörpertheorie und -optik, Friedrich-Schiller-Universität, Max-Wien-Platz 1, 07743 Jena, Germany*

(Dated: January 4, 2024)

I. GEOMETRY

Figures SM1 and SM2 show the topview of the studied heterostructures without ZLG. In order to better show the Pb layer, we have peeled off the graphene overlayer.

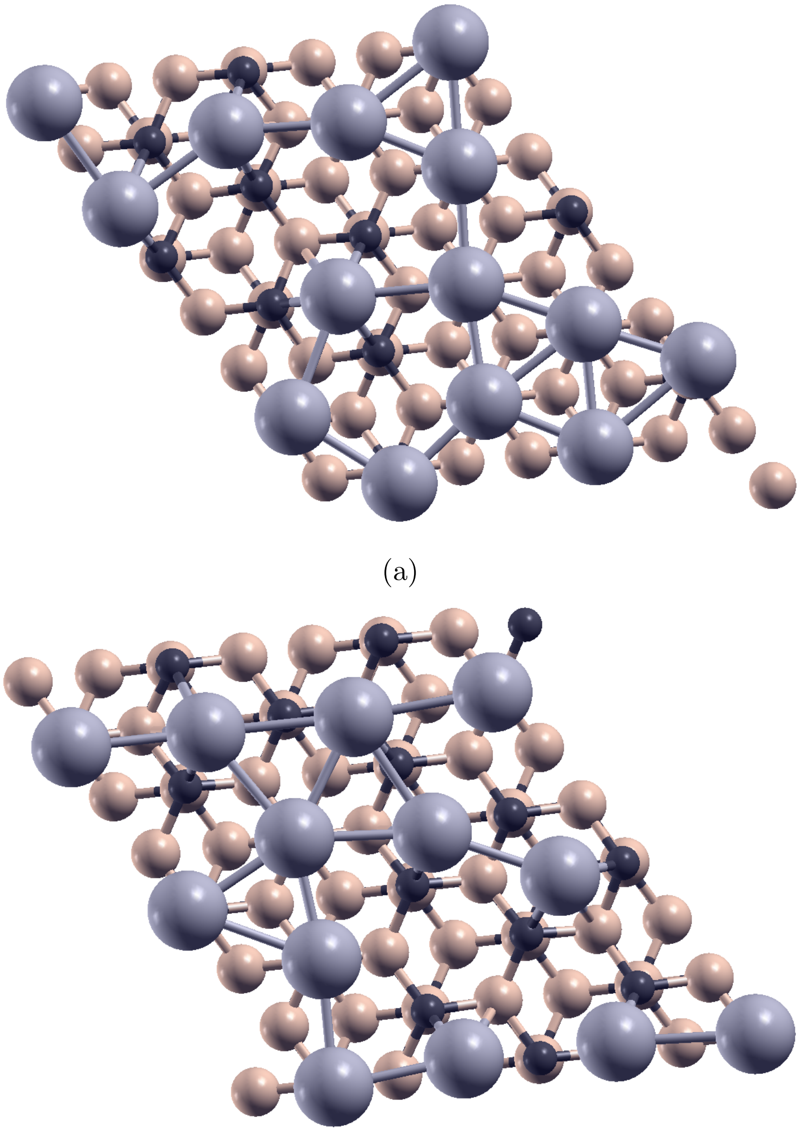

(a)

(b)

FIG. SM1: Top view of the intercalated Pb with 14 atoms (a) and 13 atoms (b) on C-terminated SiC. The uppermost graphene layer is not displayed for simplicity.

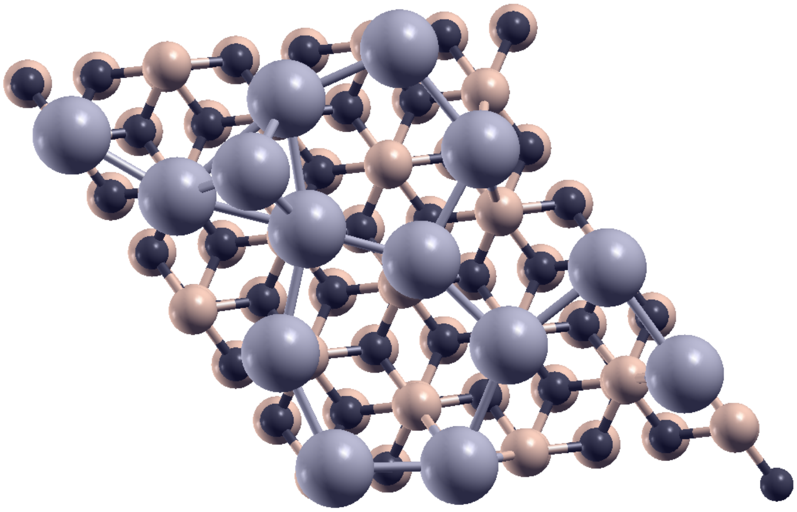

(a)

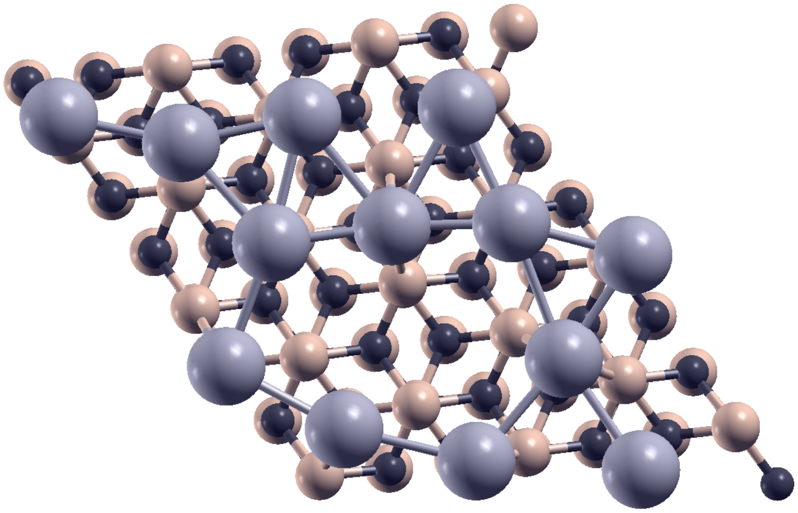

(b)

FIG. SM2: Top view of the intercalated Pb with 14 atoms (a) and 13 atoms (b) on Si-terminated SiC. The uppermost graphene layer is not displayed for simplicity.

A. Electron Charge Difference

In order to understand the reason why the Dirac cones of graphene in peeled-off Gr-Pb are shifted, the electron density difference with respect to the monolayers has been calculated. In particular, the electron charge density has been computed for graphene, plumbene and peeled-off Gr-Pb, and then the charge difference has been calculated as:

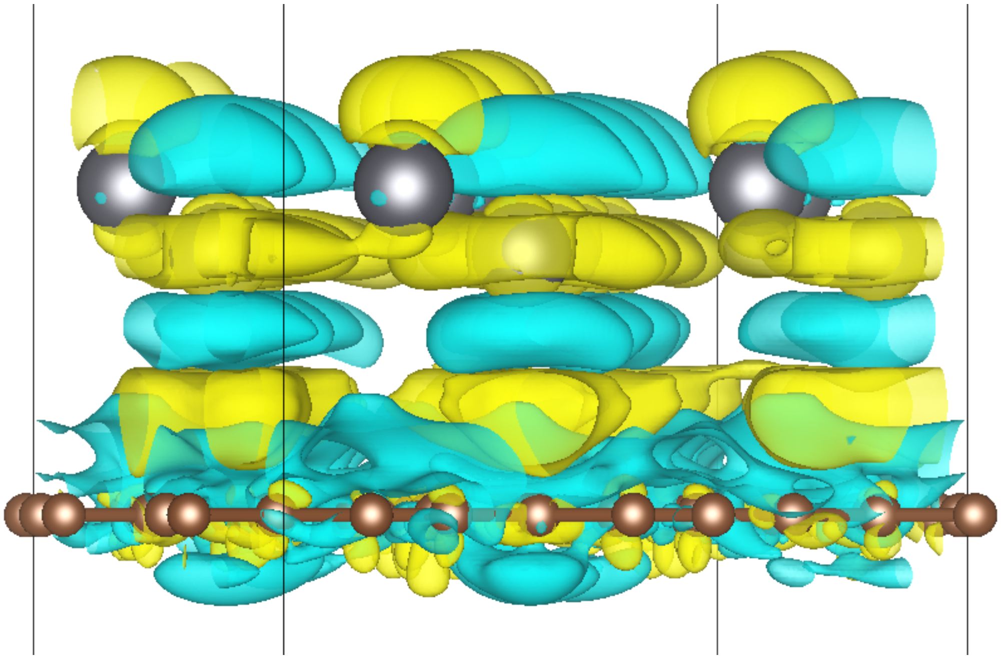

FIG. SM3: Electron charge difference between Gr-Pb peeled-off from the C-terminated SiC substrate, and the monolayers, graphene ($2\sqrt{7} \times 2\sqrt{7}$) and Pb ($\sqrt{7} \times \sqrt{7}$). Yellow color denotes a positive value of $\Delta\rho$, whereas cyan region indicate negative values of the charge difference. Large grey spheres represent Pb atoms, small brown spheres C atoms.

$$\Delta\rho = \rho_{\mathrm{PO}} - \rho_{\mathrm{graphene}} - \rho_{\mathrm{2DPb}} \qquad (1)$$

The calculated difference is shown in Fig. SM3. Even if a clear pattern of charge separation is not visible due to the mixed overlap scheme of the atom induced by the rotation angle between the layers, some charge separation domains can be observed between graphene and plumbene. This charge separation results in the presence of a vertical internal dipole in the bilayer and affects the energy dispersion of the bands, since it is known that the Dirac cones of graphene are shifted in energy in presence of a proper external electric field.
